# The Effect of Inhaled Air Particulate Matter SRM 1648a on the Development of Mild Collagen-Induced Arthritis in DBA/J Mice

**DOI:** 10.1007/s00005-022-00654-9

**Published:** 2022-07-28

**Authors:** Bernadeta Nowak, Grzegorz Majka, Małgorzata Śróttek, Anna Skałkowska, Janusz Marcinkiewicz

**Affiliations:** Chair of Immunology, Faculty of Medicine, Jagiellonian University Medical College, Krakow, Poland

**Keywords:** Air pollution, Rheumatoid arthritis, Collagen induced arthritis, Autoimmunity, Chronic inflammation

## Abstract

Air pollution is considered to be one of a risk factor for rheumatoid arthritis (RA). Collagen-induced arthritis (CIA) is commonly used as a mouse model of human RA. However, the impact of specific particulate matter (PM) components on the incidence and severity of RA has still not been established. The aim of this study was to develop an experimental model of CIA suitable to test arthritogenicity of inhaled PM. A mild form of CIA was induced in DBA1/J mice inhaled with various components of SRM 1648a PM. The incidence and severity of arthritis was assessed, and the selected serum markers of autoimmunity and inflammation were determined. Clinical arthritis was observed from the booster CII immunisation onward. Anti-cyclic citrullinated peptide antibodies, a diagnostic marker of RA, were detected in serum of these mice. All inhaled pollutants, crude PM, PM with reduced organic content, ferric, and silica nanoparticles markedly increased CIA incidence and severity. The fastest progression of CIA development was caused by crude PM and was linked to enhanced serum levels of anti-CII IgG, the prominent arthritogenic autoantibodies. On the other hand, inhaled nanoparticles enhanced serum levels of TNFα, a major proinflammatory arthritogenic cytokine. We recommend this experimental model of mild CIA to test the mechanisms of arthritis exacerbation by inhaled air pollutants. Further studies are necessary to determine whether PM-aggravated arthritis is caused by inflammatory mediators translocated from inflamed lung into systemic circulation or whether PM translocated into the bloodstream directly exacerbate joint inflammation.

**Supplementary Information:**

The online version contains supplementary material available at 10.1007/s00005-022-00654-9.

## Introduction

Rheumatoid arthritis (RA) is a systemic autoimmune disorder manifested by chronic polyarthritis. Both genetic and environmental factors are implicated in the development of the disease with highest risk conferred by genes encoding major histocompatibility complex proteins for the former (Ding et al. 2009; Raychaudhuri et al. 2012) and smoking, air pollution and exposure to silica for the latter (Hart et al. 2009; Stolt et al. 2005; Sugiyama et al. 2010).

Air pollution has long been established as a factor associated with morbidity and mortality from respiratory and cardiovascular diseases (Karimi et al. 2019; Liang et al. 2009; Pothirat et al. 2019). Furthermore, components of air pollution has been implicated in the development of autoimmune diseases such as RA and this particular link has been confirmed in several epidemiological studies (Chang et al. 2016). However, surveys attempting to establish direct association between particular pollutants and RA have not proven statistically significant relationships (De Roos et al. 2014).

It is plausible that various constituents found in particulate matter (PM)—be that organic or inorganic—might differently impact the development of RA. Our earlier in vitro studies pointed to the anticipated hyperinflammatory effect of particulate matter (both crude and devoid of organic fraction) on immune cells. However, relevant animal models must also be established in order to study how specific components of PM affect the development/exacerbation of the disease. Here, we describe our endeavour to develop a suitable in vivo/ex vivo model to elucidate the effect of particular pollutants on the disease aggravation.

The aim of this study was to adapt the murine collagen-induced arthritis (CIA) model of RA to facilitate studies on the effect of components of air pollution on the development of the disease. Original model encompasses high severity RA, thus, aggravation of the symptoms due to external factors would not be easily distinguished. Our efforts focused on reducing the score of CIA in the model without significant reduction in the disease incidence rate.

## Materials and Methods

### PM Samples

Urban particulate matter samples SRM 1648a (encoded as PM) were purchased from National Institute of Standards and Technology in the USA. The samples were composed of particulate matter collected over a period of 1 year (1976–1977) in the St. Louis (MO, USA) area into a specially designed dust collector. SRM 1648a is a conglomeration of fine and ultrafine particles with the mean particle diameter 5.85 µm. The reference material consists of high level of iron (Fe) and zinc (Zn) (transition metals) and other inorganic elements (Certificate of Analysis Standard Reference Material 1648a Urban Particulate Matter). Moreover, the SRM 1648a contains ca. 13% of carbon, including 10.5% of organic carbon. Plasma Zepto system (Diener electronic GmbH + Co. KG) has been used to eliminate organic compounds present in the reference material. Samples were treated with low-temperature plasma at highest power for 120 min. Content of carbon was determined by the elementary analysis and total organic carbon analyzer (Schimadzu, TOC-V series). The decreased carbon content from 14% in original samples (PM) to 2% in the plasma-treated PM samples (encoded as PM∆C) has been observed (Mikrut et al. 2018).

Silica nanoparticles (SiNPs) were purchased from US Research Nanomaterials, Inc (Houston, TX, USA) in the aqueous suspension (25wt%). Ferric nanoparticles (FeNPs) were obtained from PlasmaChem (Berlin, Germany) as 5% aqueous suspension. More information on the materials used to prepare the inhaled samples is available in Online Resource 1.

Before use PM and PM∆C particles were weighted on a high precision microbalance and stock suspensions water were prepared (concentrations of 1.2 mg/ml for PM, 1.3 mg/ml for PM∆C, 0.6 mg/ml for SiNPs, 0.4 mg/ml for FeNPs). The samples were sonicated for 20 min before use in each experiments.

### Mice

Inbred DBA/1 male mice were bred in the Animal Breeding Unit of Jagiellonian University College of Medicine, Krakow (Poland). Mice at 8–10 weeks of age were housed in cages with free access to standard rodent diet and water and maintained on reversed 12-h dark/12-h light cycles under clean conventional conditions in air conditioned room (22.5 ± 0.5 ℃, 50 ± 5% humidity). All animal procedures were in agreement with the guidelines from Directive 2010/63/EU of the European Parliament on the protection of animals used for scientific purposes and were approved by the Jagiellonian University Ethical Committee on Animal Experiments (approval number 16/2016).

### CIA Models

#### Model 1

DBA/1 mice (*n* = 12) were immunized intradermally with chicken sternal collagen type II (CII) (Sigma Aldrich, Germany), 100 µg/mouse in Complete Freund’s Adjuvant (CFA, 1 mg/mouse, volume of 100 µl) (Sigma Aldrich, Germany) followed with intradermal injection of CII (100 μg/mouse) in Incomplete Freund’s Adjuvant (IFA) (Sigma Aldrich, Germany) 3 weeks later (in 100 µl). Injections were made into the base of the tail of mice.

#### Model 2

DBA/1 mice (*n* = 16) were immunized intradermally with chicken sternal CII, 100 μg/mouse in saline (in 50 µl) followed with intradermal injection of CII (100 μg/mouse) in saline 3 weeks later (in 50 µl). Injections were made into the abdominal skin of mice.

#### Evaluation of arthritis

Mice were examined visually every other day for the incidence and severity of arthritis (joint swelling and redness). According to the extent of erythema and oedema of the periarticular tissues, the lesions of the four paws were each graded from 0 to 4 as follows: 0 = no swelling/normal, 1 = slight swelling of the limb or the single digits, 2 = moderate swelling/erythema of the limb and/or multiple digits, 3 = pronounced swelling and erythema, of the limb and multiple digits, 4 = severe swelling and erythema of the limb/digits and joint rigidity/deformity. Arthritis index (maximum 16 for each animal) was calculated as sum of the scores of all four paws. Paw thickness (swelling of paws) was measured using Mitutoyo micrometer. At the end of the experiment blood was collected from each mouse. Serum samples were stored at − 30 °C until assayed.

### Inhalation (PM Treatment)

**Fig. 1:**
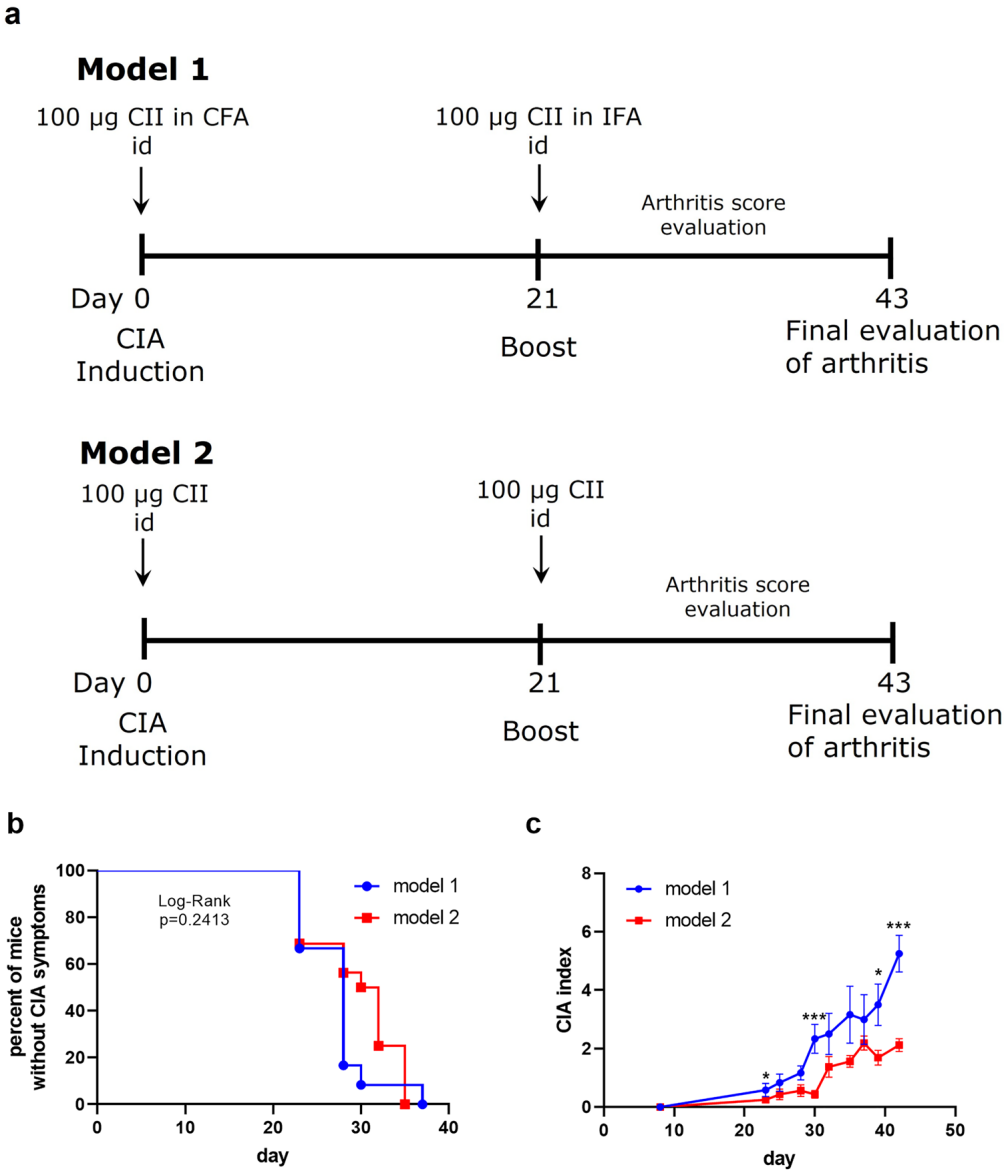
Establishment of CIA model. Model 1 (**a** upper panel): typical model for induction of CIA. Mice (*n* = 12) were immunized by intradermal (id) injection of CII in the presence of CFA (day 0) followed with boost immunization with CII in IFA (day 21). Model 2 (**a** lower panel): mice (*n* = 16) were immunized with CII in saline (without adjuvant) intradermally twice on day 0 and 21. Development of arthritis was examined by visual observation and paw thickness measurement. The percentage of mice that did not exhibit signs of arthritis in the function of time is shown (**b** model 1: blue line, model 2: red line); the severity of clinical symptoms (arthritis score) is expressed in points as CIA index (**c** model 1: blue line, model 2: red line). *P < 0.05, ***P < 0.001 denote statistically significant differences in CIA index between the models

**Fig. 2:**
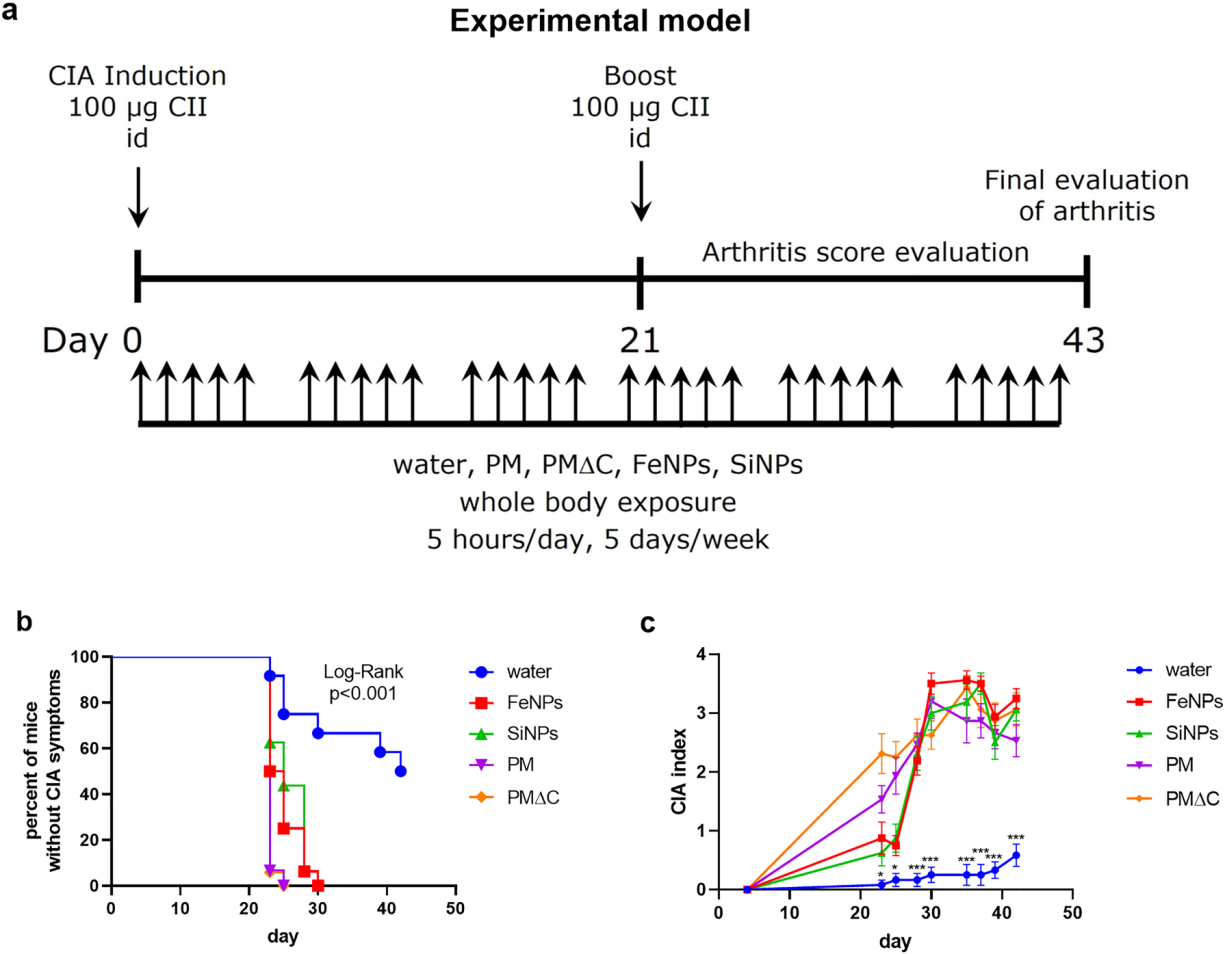
PM effect on CIA onset. Mice immunized intradermally (id) twice (day 0 and 21) with CII in saline (model 2) were inhaled with water (control mice, *n* = 12) or tested particles (PM or PMΔC or FeNPs or SiNPs, *n* = 16 each group) 5 h/day, 5 days/week for 6 weeks starting on the day of CIA induction (**a** scheme, day 0). Development of arthritis was examined by visual observation and paw thickness measurement. The percentage of mice that did not exhibit clinical symptoms of CIA in the function of time (**b**) and the severity of clinical symptoms (CIA index; **c**) is shown (water: blue line, PM: violet line, PMΔC: orange line, FeNPs: red line, SiNPs: green line). CIA index results are expressed as a mean of the measurements of each individual mouse ± SEM. *P < 0.05, ***P < 0.001 denote statistically significant differences between the control group (water) and all the tested groups (PM, PMΔC, FeNPs, SiNPs)

Mice in CIA model 2 (Fig. 1a) were treated by nebulization (Fig. 2a) 5 h per day, 5 days per week with the aqueous suspensions of PM (*n* = 16), PM∆C (*n* = 16), SiNPs (*n* = 16) or FeNPs (*n* = 16) for 6 weeks. Inhalation exposure system (Vivari, Poland) composed of DSI Mass Dosing Chamber, Mass Dosing Aerosol Controller, Nebulizer (4–6 µm) Aeroneb Prol Lab (Standard 4.0–6.0 VMD) was used. Aeroneb Pro Nebulizer (Aerogen, Galway, Ireland) was directly connected to the chamber. A whole body exposition chamber allowed free animal movement, but during the 5 h daily exposure the animals were deprived of food and water. Control mice (*n* = 12) were exposed to distilled water under conditions described above.

According to the manufacturer, the Aeroneb Pro aerosol had a mass median aerodynamic diameter of 2.1 μm. Geometric standard deviation and fine particle fraction (< 5 μm) determined by laser diffraction using the Malvern Spraytech was 2.2 and 83%, respectively. The dose concentration of obtained aerosol was 400 μg/m^3^ (daily alert level in Poland is 150 μg/m^3^).

### Determination of Serum Anti-collagen IgG Concentration

Anti-CII antibodies in serum were measured by sandwich ELISA as described previously (Kwaśny-Krochin et al. 2002). In brief, plates (Costar EIA/RIA plates, Corning Incorporated, USA) were coated overnight with native chicken CII (5 μg/ml). Mouse serum samples diluted in PBS were applied to antigen-coated wells for 1 h at room temperature followed by biotin-conjugated antibodies against mouse IgG (Sigma-Aldrich, Germany), IgG1 (MP Biomedicals, USA) or IgG2a (Southern Biotech, USA). Horseradish peroxidase-conjugated streptavidin (Vector, USA) was used as detection reagent and developed with TMB Substrate Solution (Thermo Fisher Scientific, USA). The reaction was stopped with 3 M H_2_SO_4_ (POCH, Poland). The optical density of each sample was measured at *λ* = 450 nm. The antibody levels were expressed in arbitrary ELISA units (AU) calculated from the titre of anti-CII immunoglobulins at OD = 0.1 × 10.^–2^

### Anti-cyclic Citrullinated Peptide Antibody Assay

Mouse serum was examined for the presence of antibodies against citrullinated peptides using commercially available mouse anti-cyclic citrullinated peptide antibody (ACCPA) ELISA kit (Wuhan Fine Biotech Co., China). Shortly, standard and tested samples were added to antigen precoated wells of 96-well plate followed with biotin-labelled standard antigen as detection reagent. After incubation and washing HRP-Streptavidin Conjugate was added to the wells, followed with TMB Substrate Solution. The reaction was stopped with Stop Solution. The absorbance (*λ* = 450 nm) of each serum sample was measured immediately.

### Determination of Proinflammatory Cytokines in Serum

Proinflammatory cytokines in mouse serum were measured using DB Cytometric Bead Array (CBA) mouse Th1/Th2/Th17 Cytokine Kit (DB Biosciences, USA) according to manufacturer instruction. Shortly diluted serum samples were mixed with beads coated with anti-cytokine antibodies (capture beads). Diluted mouse cytokine standards and tested serum samples were incubated with PE-detection reagent. After washing, samples resuspended in washing buffer were acquired on the flow cytometer (BD FACS Calibur) with CellQuest Pro software (BD Biosciences, USA). Data were analyzed using FCAP Array Software (BD Biosciences, USA).

### Determination of Hydroxynonenal Modified Proteins in Serum

The quantity of hydroxynonenal (HNE) modified proteins in mouse serum was measured using commercially available OxiSelect™ HNE Adduct Competitive ELISA Kit (Cell Biolabs, Inc., USA) according to the manufacturer protocol. Shortly HNE-BSA standard and mouse serum samples were added to HNE conjugate coated ELISA plates. After incubation, an anti-HNE polyclonal antibodies were added, followed with an HRP-conjugated secondary antibodies. Substrate Solution was added and the enzymatic reaction was stopped by Stop Solution. The absorbance of each well was measured immediately at *λ* = 450 nm.

### Statistical Analysis

For analysis of percentage of mice without CIA symptoms Log-Rank (Mantel-Cox) test with Bonferroni correction was employed. For the rest of the results statistical significance of differences between groups was analysed using one-way ANOVA, followed, if significant, by a Tukey’s test for post hoc comparison. Results are expressed as mean ± SEM values. A *P* value < 0.05 was considered statistically significant. Analysis was performed using Graphpad Prism v. 5.01 (GraphPad Software, Inc.).

## Results

### CIA Models

To induce arthritis mice are typically immunized with CII in the presence of CFA followed with boost injection of antigen in the presence of IFA (model 1, Fig. 1a). Such treatment results in the development of severe arthritis with high incidence rate (model 1, Fig. 1b) and high index of CIA (model 1, Fig. 1c). To study the effect of air pollution components that are considered to worsen the symptoms of arthritis we have established CIA model with high incidence rate (model 2, Fig. 1b), but very mild arthritis corresponding to a relatively low score (model 2, Fig. 1c). CIA in model 2 develops at a similar rate in comparison with model 1; analysis of the period of time without symptoms for both models did not show statistically significant differences (Fig. 1b) and the observed median period of time was comparable (28 for model 1, 31 for model 2).

### PM Effect on CIA Development

#### Clinical Symptoms

In our experimental model (model 2, Fig. 2a) clinical symptoms of arthritis were observed from the booster CII immunisation onward, in all experimental groups. However, the onset of CIA was accelerated and the clinical symptoms of disease were aggravated upon exposure of mice to the tested particles. Almost all mice (CIA incidence 94%) in PM-treated groups developed symptoms of arthritis on the 23^rd^ day of the experiment (Fig. 2b). This was much earlier than in preliminary experiment during the establishment of model 2 when arthritis symptoms were observed in most mice on the 32nd day of the experiment (Fig. 1b). The analysis of the period of time without symptoms revealed statistically significant differences between the control group (inhaled with water) and all the tested groups: FeNPs, SiNPs, PM, PMΔC. Furthermore, the median of period of time without CIA symptoms differs between the control group (42 days) and other group: FeNPs—24 days, SiNPs—25 days, PM—23 days, PMΔC—23 days. However, control mice in the experimental model (model 2, Fig. 2a) were inhaled with water. Airway humidification seemed to inhibit the development of arthritis in mice. Incidence of CIA in control group was decreased to 50% (Fig. 2b) and the score of arthritis (CIA index) was greatly reduced (Fig. 2c). This might be due to the high humidity in the exposure chamber. It has been reported by others that airways humidification reduced inflammatory responses in the lung (Jiang et al. 2015). In this context the observed effect of PM exposure on the onset of CIA was even more pronounced. As shown in Fig. 2b the rate of CIA development was fastest when mice were treated with PM and PMΔC. Most mice (90%) in these groups showed symptoms of arthritis on the 23rd day of the experiment, while at the same time in nanoparticles (SiNPs, FeNPs) treated groups only 40% of mice showed clinical symptoms of the disease. Five days later (day 28) and throughout the rest of the experiment, the CIA incidence rate was comparable in all PM treated groups (Fig. 2b). Similarly, the severity of clinical symptoms of arthritis (CIA index) was delayed in the groups of mice treated with FeNPs and SiNPs compared with PM and PMΔC treated groups (Fig. 2c).

Importantly, mice inhaled with the same PM samples (crude PM, organic-devoid PM, silica and ferric nanoparticles) for up to 16 weeks, in the model of experimental autoimmune encephalomyelitis (manuscript in preparation) as well as in the atherosclerosis model (Stachyra et al. 2022) did not develop any symptoms of joint inflammation. It indicates that PM inhalation alone is not able to induce joint inflammation but CII immunization is required.

### Serum Markers of CIA

#### Anti-CII Specific Antibodies in Mouse Serum

**Fig. 3:**
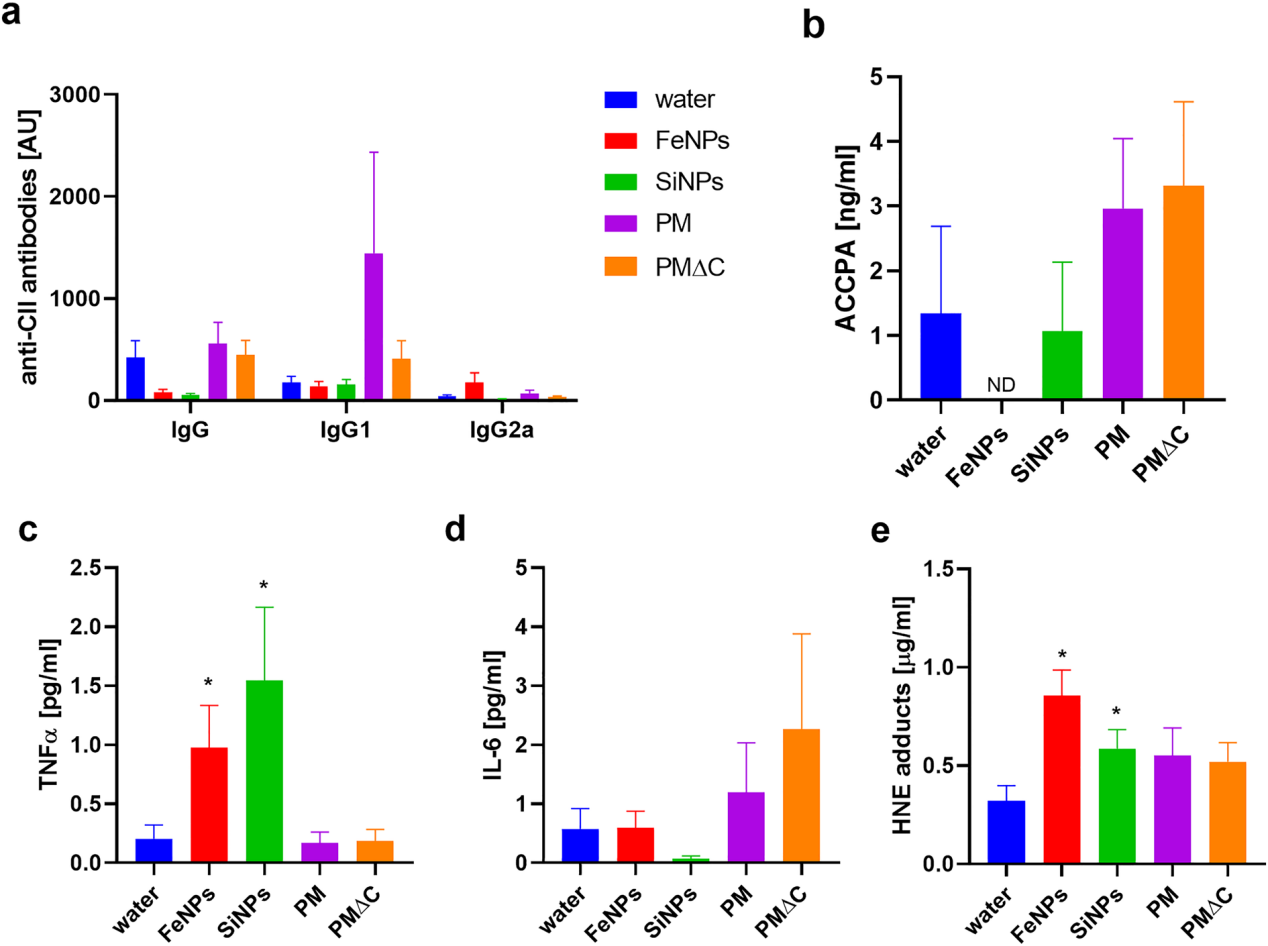
PM effect on CIA parameters in mouse serum. At the end on the experiment (day 43) blood was collected individually from each mouse immunized twice (day 0 and 21) with CII in saline, intradermally (model 2) and inhaled with water (control mice, *n* = 12; blue bars) or PM (*n* = 16, violet bars) or PMΔC (*n* = 16, orange bars) or FeNPs (*n* = 16, red bars) or SiNPs (*n* = 16, green bars) 5 h/day, 5 days/week for 6 weeks starting on the day of CIA induction (day 0). Collagen II-specific antibodies in serum (**a**) measured by ELISA are shown in arbitrary units (1 AU = serum titre at the OD = 0.1 × 10^–2^). The concentrations of ACCP antibodies (ng/ml) in serum (**b**) were measured by ELISA. Proinflammatory cytokines: TNFα (**c**) and IL-6 (**d**) were quantified by flow cytometry (CBA assay) and are expressed in pg/ml. HNE modified proteins in serum (µg/ml) were determined by competitive ELISA (**e**). All results are expressed as a mean of the measurements of each individual mouse serum ± SEM. *P < 0.05 denotes statistically significant differences between the control group (water) and all the tested groups (PM, PMΔC, FeNPs, SiNPs)

CII-specific antibodies which are generated in CIA models contribute to the pathogenesis of autoimmune arthritis. In our experimental model (model 2, Fig. 2a) the level of anti-CII antibodies in mouse serum was surprisingly low (Fig. 3a), indicating that immunization with antigen without adjuvant is not sufficient to raise adequate humoral response. The enhancement of CIA index in mice inhaled with tested particles (Fig. 2c) did not correlate with anti-CII antibody production. The levels of anti-CII antibodies (IgG, IgG1, IgG2a) in serum of control mice (inhaled with water) were very low. Anti-CII IgG amount in serum of mice treated with nano-oxides particles (FeNPs, SiNPs) was even lower that in control mice (Fig. 3a). However, the anti-CII IgG1 antibodies were elevated when mice were treated with PM, but the difference with reference to control group was not statistically significant.

#### Anti-cyclic Citrullinated Peptide Antibody

Citrullination of proteins results in formation of autoantibodies such as ACCPA. These antibodies are linked to the development of arthritis and therefore are considered as diagnostic marker of the disease. ACCPA were detected in serum of most of the tested mice. Only in mice treated with ferric oxide nanoparticles ACCPA were below detection limit of the assay. As compared to control group, mice inhaled with PM and PMΔC had notably increased amounts of ACCPA in serum, although these results were not statistically significant. The elevated amount of ACCPA correlates with faster CIA incidence rate in groups treated with PM and PMΔC (Fig. 3b).

#### Proinflammatory Cytokines in Mouse Serum

Although local joint inflammation is a hallmark of CIA, it is well known that arthritis is a systemic disease and pro-arthritic factors (e.g. proinflammatory cytokines) are present in serum. As shown in Fig. 3c in serum of mice inhaled with nano-oxides particles (FeNPs, SiNPs) TNFα levels were increased in comparison with control mice. Almost five-fold enhancement of TNFα was observed in serum of mice treated with FeNPs while SiNPs treatment resulted in almost eight-fold increase in the level of TNFα in mouse serum. PM and PMΔC did not affect TNFα production in mice, nonetheless IL-6 production was elevated two-fold by PM treatment and four-fold by PMΔC (Fig. 3d). On the other hand in mice inhaled with nano-oxide particles (FeNPs, SiNPs) the levels of IL-6 in serum were comparable to control mice.

#### HNE Modified Proteins in Serum as Oxidative Stress Marker

Arthritis and other inflammatory diseases are accompanied by oxidative imbalance in the body, which results in excess of free radicals or reactive oxygen species. One of the consequences of uncontrolled oxidative stress is the production of 4-hydroxynonenal (4-HNE), which is quantitatively one of the most important products of lipid peroxidation. What is more, HNE is highly reactive with proteins to form adduct (Castro et al. 2017). As shown in Fig. 3e the amount of HNE modified proteins in serum of mice in all tested groups (inhaled with particles) was elevated in comparison with control (water inhaled) mice. However, for groups treated with FeNPs the increase of HEN modified proteins in serum was statistically significant.

## Discussion

Several autoimmune and chronic inflammatory diseases, including rheumatoid arthritis and atherosclerosis, have been linked to the air pollution exposure. The incidence of autoimmune diseases was found to be higher in urban areas (Chiang et al. 2016; Gan et al. 2013; Hart et al. 2009, 2013). Moreover, it has been proven recently that several factors present in the air pollution particulate matter promote the development of atherosclerosis and impact the macrophage polarization towards M1 inflammatory phenotype (Stachyra et al. 2022). Therefore, it is plausible that other autoimmune processes could also be affected by inhalation of certain components of the particulate matter which would provide the explanation for the association between urbanization and incidence/exacerbation of RA. Based on in vitro studies (Gałuszka et al. 2020; Gawda et al. 2018), one may only speculate that the in vivo polarization of alveolar macrophages with particulate matter constituents will be responsible for aggravation of RA. To prove this hypothesis the suitable animal model of mild arthritis related to the human RA is needed. Widely used CIA seems to be the best candidate for evaluation of the influence of air pollution components on the progression and severity of RA (Brand et al. 2007; Pietrosimone et al. 2015).

Herein, we successfully adapted a renowned CIA model to the studies of particulate matter influence on arthritis exacerbation by employing double intradermal collagen injection (model 2) without adjuvant rather than immunization with CFA and IFA. While the incidence rate of CIA progresses similarly and remains high (> 80%) in the studied models, the severity of the disease is notably lower provided that the adjuvant is absent (model 2 vs. model 1). Therefore, anticipating the acceleration of the disease progression upon inhalation with air pollution components, model 2 was chosen for the subsequent experiments.

The second part of the study focused on elucidating the impact of inhaled air pollution components on the development of CIA. We observed a prominent shift in the onset of the disease if the animals were inhaled with particulate matter. Both crude (PM) and organic-deprived samples (PMΔC) as well as nano-oxide suspensions (FeNPs, SiNPs) significantly accelerated the progression of the disease measured by CIA incidence and severity. On day 30, mean severity index was above 3.0 in all tested groups (PM, PMΔC, FeNPs, SiNPs) compared to 0.25 in the control group. On the day of necropsy (day 42), all animals in the tested groups exhibited symptoms of CIA with mean severity index within 2.5–3.5 range, whereas in the tested group 50% of animals were affected with the mean severity index of 0.5. Furthermore, more complex samples (PM, PMΔC) had more pronounced effect on disease aggravation (measured by severity index) than simple nano-oxide suspensions (FeNPs, SiNPs). Importantly, clinical symptoms of arthritis were observed from the booster CII immunisation onward and all forms of PM tested have preponed the onset of CIA.

To our knowledge, it is the first successful experimental evidence showing the arthritogenic effect of inhaled air particulate matter on the progression of CIA. Previously, the impact of cigarette smoke and diesel exhaust particles has been evaluated in a similar model (Kang et al. 2020; Yoshino and Sagai 1999; Yoshino et al. 2002). Establishment of the mild CIA is crucial for furthers studies of the risk factors that might possibly contribute to acceleration or aggravation of rheumatoid arthritis.

Our further endeavours focused on elucidating potential mechanisms behind the exacerbation of the disease induced by inhaled particulate matter. Development of arthritis is related to both innate and autoantibody-dependent events (McInnes and Schett 2011). Accordingly, we have checked the presence of the following representative markers in the inhaled mice. Firstly, as CIA is related to the autoimmune reaction to collagen, the levels of anti-CII antibodies were determined. Secondly, the presence of ACCPA being another marker of rheumatoid arthritis has been evaluated consequently (Darrah and Andrade 2018). Moreover, the non-specific inflammatory response potentially leading to aggravation of the disease might be associated with the prolonged oxidative stress and/or the shift in immune cells polarization towards M1 phenotype (Mateen et al. 2016; Yang et al. 2020). Therefore, we have also tested the serum concentrations of HNE adducts (product of lipid peroxidation) as well as pro-inflammatory cytokine concentrations.

Crude PM samples had a notable impact on the levels of anti-CII antibodies (both IgG1 and whole IgG class) which implicates their direct impact on driving the autoimmune reactions. At the same time, they led to an increase in IL-6 serum concentration which might imply involvement of either B cells or IL-6-PAD4 axis (Yahagi et al. 2019). However, involvement of protein citrullination was not confirmed as the ACCPA levels remained unaltered upon PM inhalation. This observation confirms the opinion that the arthritogenic impact of antibodies against citrullinated proteins remains unclear (Kuhn et al. 2006). We also have not observed significant effect of PM on lipid peroxidation what may suggest their weak oxidative properties.

Particulate matter with reduced organic content (PMΔC) did not have a significant effect on anti-CII antibody levels. Surprisingly, serum ACCPA levels in this group were slightly elevated but no significant differences were found. Citrullination status could be further impacted by PMΔC judging by its positive influence on IL-6 concentration which might affect both differentiation of B cells and activity of PADs enzymes.

Ferric nano-oxide samples exerted negative impact on the serum levels of ACCPA and anti-CII antibodies. It seems plausible that aggravation of the symptoms in this group stems from the influence of pro-inflammatory cytokines such as TNFα which has long been implicated in the development and exacerbation of RA (Brennan and McInnes 2008; McInnes and Schett 2007). Importantly, TNFα is major cytokine produced by M1 macrophages. Thus, hyperinflammatory response and oxidative stress (implicated by increased levels of HNE adducts) arising after inhalation of FeNPs might have a significant impact on the visible symptoms of the disease.

Silica nano-oxide samples affected neither the anti-CII antibodies nor the ACCPA levels compared to the control group. Similarly to FeNPs, they induced a significant increase in the TNFα concentrations, which is probably responsible for promoting non-specific inflammatory response accelerating in turn the progression of rheumatoid arthritis. We also did not observe any changes in the lipid peroxidation status upon inhalation with SiNPs.

Interestingly, the visible symptoms of arthritis have not correlated with the anti-collagen antibodies concentrations in serum. Possibly, air pollution does not perpetuate the autoimmune processes per se but drives the hyperinflammatory response which contributes to development of CIA symptoms. This hypothesis is supported by the increased concentrations of proinflammatory cytokines: TNFα in nano-oxide groups, the major arthritogenic cytokine, and IL-6 in PM and PMΔC groups (Brennan and McInnes 2008).

We have managed to adapt the well-established model to study the effect of inhaled particles on rheumatoid arthritis. All the tested samples, crude particulate matter, particulate matter with reduced organic content, silica and FeNPs—had a significant accelerating and aggravating effect on the symptoms of the disease (paw swelling). Our further studies focused on elucidation of the underlying mechanisms which seem to be different for two groups of samples. More studies are necessary to confirm the preliminary results which point to complex samples—PM, PMΔC—seemingly affecting the autoimmune process and IL-6-PAD4 axis at the same time, whereas nano-oxide suspensions—SiNPs, FeNPs—induce massive upregulation of TNFα implicating the M1 polarization as a potential pathway for aggravation of joint inflammation. The effect of the latter has recently been confirmed in arthritis model including inhalation with the very same suspensions of nano-oxides (Stachyra et al. 2022). Our previous study confirms these data. We have shown that exposure of macrophages to low concentrations of PM may prime the cells to hyperinflammatory response (M1-type response) upon contact with LPS (Gawda et al. 2018).

**Fig. 4:**
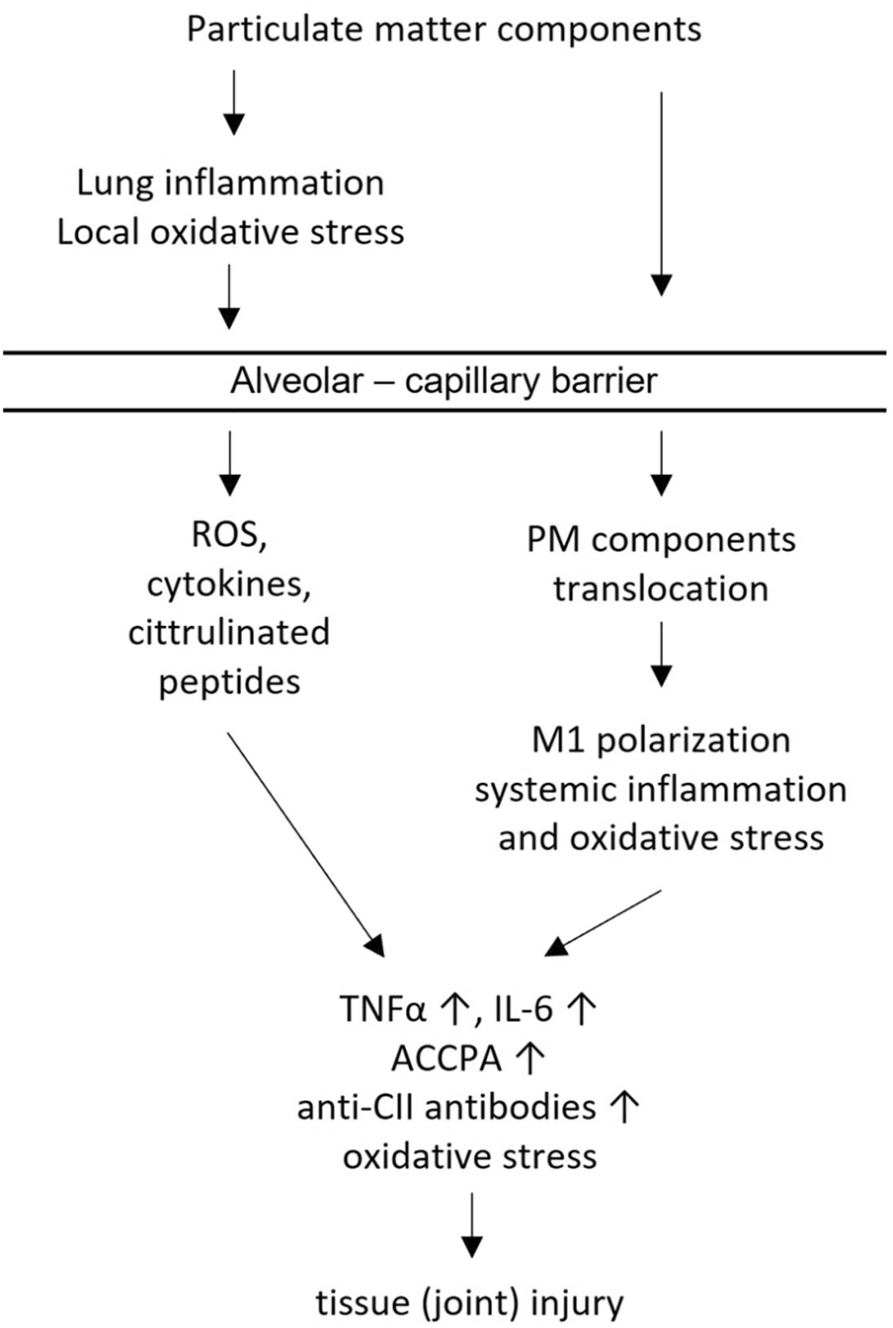
Possible mechanisms governing particulate matter components impact on the joint inflammation and injury in rheumatoid arthritis

It is important to realize the limitations of the presented study. Employing the whole body exposure setting allows for reduction of stress of the inhaled animals and does not restrain their movement. However, measurement of the effective exposure dose is not feasible in such system since the inhaled compounds are not being delivered directly to the animals’ respiratory tract. What is more, parameters measured in the murine serum (anti-CII antibodies, ACCPA, HNE adducts, cytokines) come from the serum collected upon sacrifice. Therefore, we are only able to compare the end point status of the studied animals while some of the changes might be happening earlier during the development of the disease. In spite of these limitations, herein we may present the model of mild CIA, suitable for the investigation of mechanisms of RA exacerbation by inhalation of various air pollutants. However, further studies on deposition and retention of inhaled particulate matter in the respiratory tract and their possible transport into the blood circulation (translocation) are necessary. Such investigations will provide a key information about the target organs (cells) of inhaled particles. Hypothetically, air pollution exposure may have either a direct effect on local joint inflammation or an indirect effect linked to the lung-derived systemic inflammation (Fig. 4). This hypothesis is in agreement with previous studies showing the reciprocal association of respiratory tract/lung inflammation with arthritis (Ford et al. 2020; Kronzer et al. 2022).

Finally, we suggest the following scenario of impact of PM inhalation on the development of CIA: immunisation with CII stimulates the generation of anti-CII-IgG. Interaction of cartilage CII antigen with specific antibodies induces joint inflammation characterized by an increased local capillary permeability. The local endothelium becomes the *locus minoris resistentiae*. Concurrent PM inhalation induces lung inflammation followed by the leak of inhaled nanoparticles and/or generated inflammatory mediators (e.g. TNFα) into circulation. Furthermore, these agents may be translocated from the blood stream into already inflamed joints and are responsible for the aggravation of arthritis. Nevertheless, antibodies to CII are a prerequisite for CIA as well as for rheumatoid arthritis (Gan et al. 2013).

## Supplementary Information

Below is the link to the electronic supplementary material.

Supplementary file1 (PDF 68 KB)
