# Supplementary material for: The Effect of Inhaled Air Particulate Matter SRM 1648a on the Development of Mild Collagen-Induced Arthritis in DBA/J Mice
Source: Arch Immunol Ther Exp (Warsz). 2022 Jul 28;70(1):17. doi: 10.1007/s00005-022-00654-9 (PMC9334371; doi:10.1007/s00005-022-00654-9)
Supplement: Supplementary file 1 — Supplementary file1 (PDF 68 KB) [file 5_2022_654_MOESM1_ESM.pdf]

PM samples origin and compostion

Urban PM was purchased from the National Institute of Standard and Technology US (SRM 1648a). PM was treated with cold plasma for 120 minutes in order to obtain PMΔC. The

	C	H	N	S
PM	14.10	2.41	3.12	4.96
PMΔC	1.87	1.20	0.83	6.18

Both PM and PMΔC contain various other elements such as: chlorine, potassium, calcium, titanium, vanadium, chromium, manganese, iron, nickel, copper, zinc, bromine, rubidium, strontium and lead. PM contains ca 14.10% of carbon including 10.5% organic carbon. The carbon content is reduced to less than 2% by plasma treatment (PMΔC). Additional information on PM and PMΔC composition can be found in: Mikrut M, Regiel-Futyra A, Samek L, Macyk W, Stochel G, van Eldik R (2018) Generation of hydroxyl radicals and singlet oxygen by particulate matter and its inorganic components. Environmental Pollution 238:638-646.

Iron (III) oxide produced by chemical synthesis with average particle size of 4-8 nm was purchased from PlasmaChem as a 5% aqueus suspension (cat. no PL-FeO). The composition and purity was confirmed by the producer with PCS particle size analysis, UV-VIS spectroscopy, Raman spectroscopy and TEM analysis.

Silica oxide produced by laser gas phase synthesis was purchased from US Research Nanomaterial (cat no. US3437) with the following Certificate of Analysis: SiO2 – 99%, Cu – 85 ppm, Co – 12 ppm, Ti – 65 ppm, Ni – 65 ppm, K – 66 ppm, Ca – 70 ppm, Mg – 5 ppm, Al – 32 ppm, Bi – 6 ppm, Na – 30 ppm, P – 9 ppm, Mn – 18 ppm, Fe – 20 ppm.
